# Predictors of Positive Bronchoscopy in Children with Suspected Foreign Body Aspiration: A Retrospective Cross-Sectional Study

**DOI:** 10.3390/children13070963

**Published:** 2026-07-21

**Authors:** Şule Demir, Mehmet Latif Özdemir, Arda Kaya, Murat Ayar, Gizem Beril Özdemir, Avni Merter Keçeli, Aykut Çağlar

**Affiliations:** 1 Department of Pediatric Emergency Care, Faculty of Medicine, Aydın Adnan Menderes University, 09100 Aydın, Turkey; aykut.caglar@adu.edu.tr; 2 Department of Pediatrics, Faculty of Medicine, Aydın Adnan Menderes University, 09100 Aydın, Turkey; m.latif.ozdemir@adu.edu.tr (M.L.Ö.); m.ayar@adu.edu.tr (M.A.); 3 Department of Pediatric Surgery, Faculty of Medicine, Aydın Adnan Menderes University, 09100 Aydın, Turkey; ardakaya@adu.edu.tr (A.K.); gbozdemir@adu.edu.tr (G.B.Ö.); 4 Department of Radiology, Division of Pediatric Radiology, Faculty of Medicine, Aydın Adnan Menderes University, 09100 Aydın, Turkey; avni.merter.keceli@adu.edu.tr

**Keywords:** air trapping, bronchoscopy, chest radiography, foreign body aspiration, pediatric airway

## Abstract

**Highlights:**

**What are the main findings?**

- Air trapping on chest radiography showed the strongest association with foreign body detection during bronchoscopy, although the estimated effect was imprecise.
- Nearly one-quarter of children with bronchoscopically confirmed foreign body aspiration had normal chest radiographs, indicating that normal radiographic findings do not exclude the diagnosis.

**What are the implications of the main findings?**

- Clinical decisions regarding bronchoscopy should integrate patient history, physical examination, and radiographic findings rather than rely on any single predictor.
- These findings should be interpreted within the selected population of children who had already undergone bronchoscopy.

**Abstract:**

Objective: Foreign body aspiration (FBA) is a common pediatric emergency, and bronchoscopy remains the diagnostic and therapeutic gold standard despite its invasive nature and associated procedural risks. This study aimed to evaluate the clinical and radiographic factors associated with foreign body detection during bronchoscopy in children undergoing bronchoscopy for suspected FBA. Methods: This single-center, retrospective, cross-sectional study included children aged 0–18 years who underwent bronchoscopy for suspected FBA at a tertiary pediatric emergency department between January 2010 and January 2026. Of 120 eligible patients, 25 were excluded because of incomplete medical records, resulting in a final cohort of 95 children. Predictors of bronchoscopy positivity were evaluated using Firth’s penalized logistic regression because of outcome imbalance and separation in some radiographic variables. Results: The median age was 19.0 months (interquartile range [IQR] 14.0–26.0), and 55 patients (57.9%) were male. Foreign bodies were identified in 81 children (85.3%), whereas bronchoscopy was negative in 14 (14.7%). Air trapping on chest radiography showed the strongest association with foreign body detection during bronchoscopy (adjusted OR 33.80, 95% CI 4.19–4389.05; *p* < 0.001), although the wide confidence interval indicates substantial statistical uncertainty. A normal chest radiograph was associated with a lower likelihood of foreign body detection (OR 0.10, 95% CI 0.02–0.34; *p* < 0.001). Nevertheless, normal radiographic findings did not exclude FBA, as 24.7% of children with bronchoscopically confirmed FBA had normal chest radiographs. Conclusions: Among children undergoing bronchoscopy for suspected FBA, radiographic air trapping showed the strongest association with foreign body detection, although this finding should be interpreted cautiously because of substantial statistical uncertainty. A normal chest radiograph could not reliably exclude FBA, and individual clinical findings showed limited discriminatory value. These findings apply only to children who had already been selected for bronchoscopy and support integrating clinical history, physical examination, and imaging findings when evaluating such patients.

## 1. Introduction

Foreign body aspiration (FBA) remains an important pediatric emergency and continues to be associated with substantial morbidity and potential mortality, particularly in infants and young children [1,2]. The highest risk is generally observed in children younger than three years of age, reflecting age-related anatomical, developmental, and behavioral factors, including immature swallowing coordination, narrow airways, and frequent oral exploration of objects [1,2,3]. Because delayed recognition may lead to airway obstruction, recurrent respiratory symptoms, atelectasis, pneumonia, or life-threatening respiratory compromise, timely diagnosis and appropriate bronchoscopic management remain essential [2,4].

Diagnosing foreign body aspiration can be challenging because clinical manifestations are often variable and nonspecific. Although a witnessed aspiration event, sudden coughing episode, choking, wheezing, or respiratory distress may increase clinical suspicion, these findings are not consistently present in all children, and their absence does not exclude the diagnosis [2,4,5]. Chest radiography is routinely used as the initial imaging modality in children with suspected FBA; however, radiographic abnormalities may be absent, particularly in cases involving radiolucent foreign bodies [5,6,7]. Consequently, normal chest radiographs cannot reliably exclude FBA and may contribute to diagnostic uncertainty and delayed diagnosis, frequently requiring bronchoscopic evaluation despite inconclusive radiographic findings [5,6,7].

Rigid bronchoscopy remains the gold standard for the diagnosis and treatment of FBA in children [4,5]. However, bronchoscopy is an invasive procedure that requires general anesthesia and may be associated with procedure-related complications, increased healthcare costs, and unnecessary interventions in patients without an aspirated foreign body [5,6]. Despite advances in imaging and clinical assessment, reported rates of negative bronchoscopy remain substantial across pediatric series, reflecting the ongoing difficulty of accurately identifying children who truly require endoscopic evaluation [2,6,7]. Although several clinical and radiographic findings have been proposed as predictors of foreign body aspiration, their diagnostic performance remains inconsistent across studies, and the optimal approach to patient selection for bronchoscopy remains uncertain [4,5,7].

Therefore, the aim of this study was to evaluate the clinical and radiographic factors associated with foreign body detection during bronchoscopy in children with suspected FBA. We also sought to determine the rate and characteristics of negative bronchoscopy findings in a tertiary pediatric emergency department. By identifying factors associated with bronchoscopy positivity, we aimed to provide evidence that may contribute to clinical decision-making in children undergoing bronchoscopy for suspected FBA.

## 2. Materials and Methods

### 2.1. Study Design and Population

**Figure 1:**
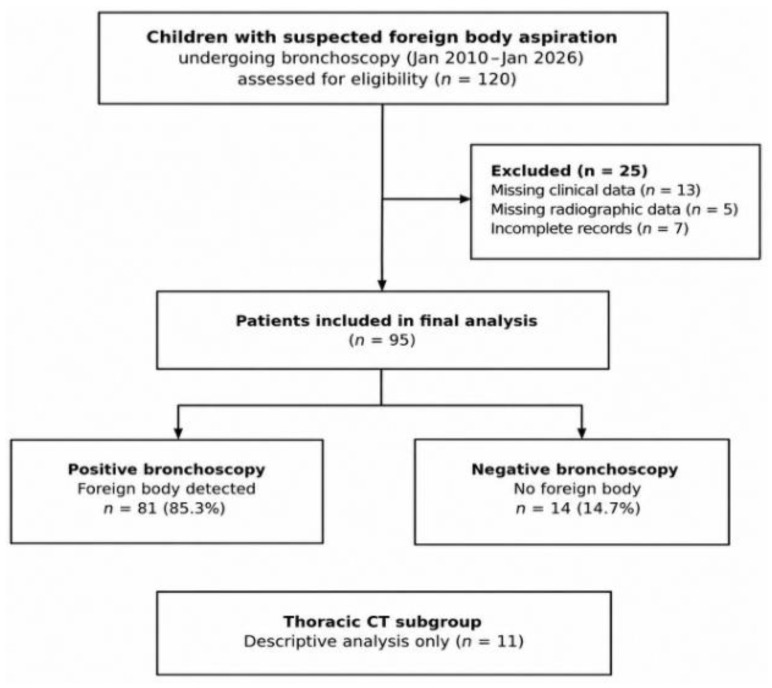
Patient selection flow diagram. CT findings were analyzed descriptively owing to the small subgroup size. FBA, foreign body aspiration; CT, computed tomography.

This was a single-center, retrospective, cross-sectional study conducted at a tertiary pediatric emergency department. Following approval by the institutional ethics committee, the medical records of all children aged 0–18 years who presented with suspected foreign body aspiration (FBA) and underwent bronchoscopy between 1 January 2010 and 1 January 2026 were retrospectively reviewed for research purposes. Patients with incomplete medical records or missing key study variables were excluded. During the study period, 120 patients underwent bronchoscopy for suspected FBA; 25 were excluded because of incomplete data, resulting in a final analytic cohort of 95 patients (Figure 1).

Children with suspected foreign body aspiration were initially evaluated in the pediatric emergency department and subsequently assessed by the pediatric surgery team, which made the final decision regarding bronchoscopy based on the overall clinical presentation, physical examination findings, and radiographic assessment. All bronchoscopies were performed using rigid bronchoscopy by members of the pediatric surgery team according to routine institutional clinical practice. Although no formal written institutional protocol was in place, clinical decision-making and procedural management followed the routine clinical practice of the pediatric surgery team throughout the study period.

Demographic data (age, sex), clinical presentation (symptom duration, delayed presentation, aspiration history, sudden coughing episode, cyanosis/choking history), physical examination findings (wheezing, dyspnea, retraction, oxygen saturation), radiographic findings, thoracic computed tomography (CT) findings, and bronchoscopy results were extracted from medical records using a standardized data collection form. Chest radiographic findings were extracted retrospectively from the medical records based on the original radiology reports and documented clinical records. The images were not reinterpreted for the purposes of this study. Thoracic CT was not performed according to a predefined study protocol. Instead, the decision to obtain CT was made at the discretion of the treating physicians based on the individual clinical presentation and diagnostic uncertainty. The primary outcome was foreign body detection during bronchoscopy.

### 2.2. Statistical Analysis

All analyses were performed using R version 4.6.0 (R Foundation for Statistical Computing, Vienna, Austria). Continuous variables are presented as median (interquartile range [IQR]) and categorical variables as counts and percentages. Comparisons between patients with positive and negative bronchoscopy findings were performed using the Mann–Whitney U test, Pearson chi-squared test, or Fisher’s exact test, as appropriate.

Predictors of foreign body detection were evaluated using Firth’s penalized logistic regression because of the small number of negative bronchoscopy outcomes and separation in some radiographic variables. Odds ratios (ORs) and 95% confidence intervals (CIs) were estimated, with confidence intervals derived from the profile penalized likelihood. Because of the limited number of negative bronchoscopy outcomes (*n* = 14), the multivariable model was restricted to two predictors to avoid overfitting and was reported as a sensitivity analysis only. The model was intended to explore associations between prespecified variables and bronchoscopy positivity and was not developed or validated as a clinical prediction tool. Predictors were selected on the basis of clinical relevance rather than univariable *p*-values: air trapping on chest radiography was retained as the strongest radiographic predictor, with wheezing included as an a priori respiratory covariate. Normal chest radiography was not included in the multivariable model because air trapping was selected as the primary radiographic variable based on its greater clinical relevance. In addition, the model was intentionally restricted to two prespecified variables owing to the limited number of negative bronchoscopy outcomes. Similarly, cyanosis/choking history was not included in the multivariable model despite reaching statistical significance in the univariable analysis because of its reliance on caregiver recall, which is susceptible to recall bias, and because the model was restricted to two predictors given the limited number of negative bronchoscopy outcomes.

The negative bronchoscopy rate was reported with an exact binomial (Clopper–Pearson) 95% confidence interval. CT findings were analyzed descriptively because only 11 patients underwent CT. All statistical tests were two-sided, and *p* < 0.05 was considered statistically significant. Reporting followed the STROBE statement.

## 3. Results

A total of 95 children who underwent rigid bronchoscopy for suspected foreign body aspiration were included in the study. The median age was 19.0 months (IQR 14.0–26.0), and 55 patients (57.9%) were male. A history of witnessed aspiration was reported in 88 patients (92.6%), and a sudden coughing episode was reported in 89 (93.7%). The median symptom duration before presentation was 25 h (IQR 4–48), and delayed presentation (>24 h) occurred in 50 patients (52.6%). The demographic and clinical characteristics of the study population are summarized in Table 1.

On chest radiography, air trapping was observed in 46 patients (48.4%), atelectasis in 7 (7.4%), and consolidation in 17 (17.9%), whereas 31 patients (32.6%) had normal radiographs. Thoracic computed tomography was performed in 11 patients (11.6%). Among the nine patients with positive bronchoscopy, the most frequent CT findings were a direct foreign body sign (*n* = 6) and consolidation (*n* = 6), followed by atelectasis (*n* = 3) and air trapping (*n* = 2). Detailed CT findings are presented in Supplementary Table S2.

Foreign bodies were identified during bronchoscopy in 81 of 95 patients (85.3%), while bronchoscopy was negative in 14 patients (14.7%). The clinical and radiographic characteristics of patients with negative bronchoscopy findings are presented in Supplementary Table S1. Among positive cases, the most common location was the right main bronchus (65.4%), followed by the left main bronchus (23.5%), trachea (4.9%), and other bronchial locations (6.2%). Organic foreign bodies accounted for 98.8% of all retrieved materials. No bronchoscopy-related complications were observed.

Comparisons between patients with positive and negative bronchoscopy findings are presented in Table 2. Age, symptom duration, sex, delayed presentation, aspiration history, sudden coughing episode, wheezing, dyspnea, retractions, atelectasis, consolidation, concomitant infection, and chronic disease status did not differ significantly between groups (all *p* > 0.05). Air trapping on chest radiography was more common among patients with a confirmed foreign body than among those with negative bronchoscopy findings (56.8% vs. 0%, *p* < 0.001). In contrast, a normal chest radiograph was present in 24.7% of children with a confirmed foreign body and in 78.6% of those with negative bronchoscopy findings (*p* < 0.001).

The results of the univariable and multivariable Firth’s penalized logistic regression analyses are presented in Table 3. In univariable analyses, air trapping on chest radiography was associated with a positive bronchoscopy result (OR 37.99, 95% CI 4.79–4914.56, *p* < 0.001), whereas a normal chest radiograph was associated with a lower likelihood of foreign body detection (OR 0.10, 95% CI 0.02–0.34, *p* < 0.001). Cyanosis/choking history was also associated with bronchoscopy outcome (OR 0.28, 95% CI 0.09–0.93, *p* = 0.038). No other clinical variables reached statistical significance.

A multivariable Firth’s penalized logistic regression model including radiographic air trapping and wheezing was subsequently constructed. Air trapping remained independently associated with foreign body detection during bronchoscopy (adjusted OR 33.80, 95% CI 4.19–4389.05, *p* < 0.001), whereas wheezing was not independently associated with bronchoscopy positivity (adjusted OR 1.32, 95% CI 0.30–7.81, *p* = 0.728).

## 4. Discussion

In this retrospective, cross-sectional study of children undergoing bronchoscopy for suspected foreign body aspiration, foreign bodies were identified in 85.3% of cases, whereas 14.7% of bronchoscopies were negative. Air trapping on chest radiography was strongly associated with foreign body detection during bronchoscopy, although the estimated effect should be interpreted cautiously because of substantial statistical uncertainty. In contrast, nearly one-quarter of children with a confirmed foreign body had normal chest radiographs, highlighting the limited ability of radiography alone to exclude the diagnosis. Importantly, because only children who underwent bronchoscopy were included, our findings identify factors associated with bronchoscopy positivity within an already selected population rather than predictors of foreign body aspiration among all children presenting with suspected aspiration. These findings emphasize the ongoing diagnostic challenges of pediatric FBA and support the need for careful integration of clinical and radiographic findings when evaluating children who have already been selected for bronchoscopic assessment.

The bronchoscopy positivity rate observed in our cohort (85.3%) was relatively high and is consistent with recent pediatric studies reporting foreign body detection rates ranging from approximately 70% to 90% among carefully selected patients undergoing bronchoscopy [2,4,5]. This relatively high positivity rate may also reflect our institutional referral pathway, in which the final decision to perform bronchoscopy was made by the pediatric surgery team after considering the overall clinical presentation, physical examination findings, and radiographic assessment. Conversely, 14.7% of bronchoscopies in our series were negative. Although negative bronchoscopy rates vary considerably across studies, a certain proportion of negative procedures is generally considered acceptable given the potentially serious consequences of missed foreign body aspiration [6,7]. Nevertheless, negative bronchoscopies expose children to unnecessary general anesthesia, procedural risks, and additional healthcare costs. Recent investigations have therefore emphasized the importance of improving patient selection and identifying reliable clinical and radiological predictors that can support decision-making while minimizing unnecessary invasive procedures [5,6,7]. However, improving patient selection should never come at the expense of delaying or missing the diagnosis of foreign body aspiration. In children with persistent clinical suspicion, bronchoscopy remains the preferred diagnostic and therapeutic approach despite the possibility of negative findings.

One of the most clinically relevant findings of our study was the strong association between radiographic air trapping and foreign body detection during bronchoscopy. Air trapping was significantly more common among children with confirmed foreign bodies and remained independently associated with bronchoscopy positivity in multivariable analysis. However, the adjusted effect estimate was accompanied by a very wide confidence interval, reflecting the limited number of negative bronchoscopy cases and near-complete separation despite the use of Firth’s penalized logistic regression. Therefore, the magnitude of this association should be interpreted cautiously and considered exploratory until confirmed in larger studies. This observation is consistent with recent studies identifying unilateral hyperinflation or air trapping as one of the most valuable radiographic indicators of airway foreign bodies in children [5,7]. However, our findings also highlight the limitations of chest radiography. Nearly one-quarter of children with a confirmed foreign body had normal chest radiographs, emphasizing that the absence of radiographic abnormalities cannot reliably exclude FBA. Similar observations have been reported in recent pediatric studies and systematic reviews, which demonstrated that a substantial proportion of children with bronchoscopically confirmed foreign bodies may present with normal radiographic findings, particularly when radiolucent organic materials are aspirated [7,8,9,10]. Therefore, chest radiography should be considered a complementary diagnostic tool rather than a definitive rule-out test, and persistent clinical suspicion should continue to guide decisions regarding bronchoscopic evaluation.

In contrast to radiographic air trapping, most clinical variables evaluated in our study were not significantly associated with foreign body detection. Although a witnessed aspiration event and sudden coughing episode were highly prevalent among children with confirmed foreign bodies, these findings were also common among children with negative bronchoscopy results. Likewise, wheezing, dyspnea, and retractions did not significantly differentiate between groups. Previous studies have similarly reported that individual clinical findings often demonstrate limited diagnostic accuracy when considered in isolation and should not be used as the sole basis for bronchoscopy decisions [11,12,13]. The heterogeneous clinical presentation of FBA may be influenced by the type, size, location, and duration of the aspirated material, as well as by variability in caregiver-reported history. Therefore, a comprehensive assessment that integrates clinical history, physical examination, and imaging findings is likely to provide greater diagnostic value than any single clinical feature alone.

The role of thoracic computed tomography in the evaluation of suspected FBA remains controversial. Recent studies and systematic reviews have demonstrated excellent sensitivity and negative predictive value for CT, suggesting that it may reduce the number of unnecessary bronchoscopies in selected patients [6,7,9,14]. However, concerns regarding radiation exposure, cost, availability, and the potential need for sedation continue to limit its routine use in children. Recent evidence has also suggested that chest radiography may retain substantial diagnostic value despite its lower sensitivity compared with CT. In a bronchoscopy-based cohort study, Sarac and Yazici reported that chest radiography was not inferior to CT with respect to overall diagnostic F1 score, supporting its continued role as the first-line imaging modality in children with suspected FBA [15]. In our cohort, CT was performed in only a small proportion of patients and therefore could not be formally evaluated as a diagnostic predictor. Nevertheless, our findings support current evidence suggesting that CT may be particularly useful in children with persistent clinical suspicion despite inconclusive chest radiography, while bronchoscopy should remain the definitive diagnostic and therapeutic procedure when the probability of FBA is high [7,9,14].

The limitations of individual clinical and radiographic findings have prompted increasing interest in integrated diagnostic algorithms for children with suspected FBA. Recent studies have shown that combining elements of caregiver-reported history, physical examination findings, and imaging results improves diagnostic performance compared with reliance on any single variable alone [11,12,13]. In particular, clinical prediction models incorporating choking history, abnormal respiratory examination findings, and radiographic abnormalities have demonstrated promising accuracy in identifying children at high risk for airway foreign bodies while potentially reducing unnecessary bronchoscopic procedures [12,13]. However, despite these encouraging results, no clinical prediction model has yet undergone sufficient external validation to support widespread implementation in routine practice [16]. Our findings support this concept. Although most individual clinical variables were not significantly associated with bronchoscopy positivity, radiographic air trapping emerged as a strong predictor, suggesting that diagnostic decisions should be based on the overall pattern of findings rather than isolated symptoms or signs. Future prospective multicenter studies are needed to externally validate integrated prediction models and optimize patient selection for bronchoscopy in children with suspected FBA [16].

## 5. Conclusions

In this study of children undergoing bronchoscopy for suspected foreign body aspiration, radiographic air trapping showed the strongest association with foreign body detection during bronchoscopy. However, because the estimated effect was accompanied by a very wide confidence interval, this finding should be interpreted cautiously and confirmed in larger studies. In contrast, normal chest radiography did not reliably exclude the diagnosis, and most clinical findings showed limited discriminatory value when evaluated individually. These findings suggest that no single clinical or radiographic feature is sufficient to confirm or exclude foreign body aspiration. Instead, our findings support a diagnostic approach that integrates clinical history, physical examination, and imaging findings when evaluating children who have already been selected for bronchoscopy. Bronchoscopy remains the definitive diagnostic and therapeutic modality in children with persistent suspicion of foreign body aspiration.

## 6. Strengths and Limitations

This study has several strengths, including the use of bronchoscopy-confirmed outcomes, a relatively long study period, and the evaluation of multiple clinical and radiographic predictors of foreign body aspiration. In addition, Firth’s penalized logistic regression was used to address outcome imbalance and separation issues.

However, several limitations should be considered. The retrospective, single-center design may limit the generalizability of the findings. In addition, although bronchoscopy decisions were consistently made by the same pediatric surgery team throughout the study period, the long study period may still have introduced temporal variability in diagnostic imaging and overall clinical practice, which could have influenced the study findings. The relatively small number of negative bronchoscopy cases (*n* = 14) limited statistical power and resulted in near-complete separation for some radiographic variables, particularly air trapping, which was absent in all negative bronchoscopy cases. Although Firth’s penalized logistic regression was used to address this issue, the resulting confidence intervals for air trapping and normal chest radiography remained extremely wide, and these estimates should therefore be interpreted with caution despite their statistical significance. In addition, thoracic CT was available in only a limited number of patients. Moreover, because of the retrospective design, the specific types of aspirated foreign bodies were not consistently documented in the medical records, precluding a reliable subgroup analysis. The exclusion of 25 patients because of incomplete medical records may also have introduced selection bias, which is an inherent limitation of retrospective studies. Furthermore, because only children who underwent bronchoscopy were included, the findings identify factors associated with bronchoscopy positivity within an already selected population and therefore cannot be generalized to all children presenting with suspected foreign body aspiration and cannot be used to determine which patients should undergo bronchoscopy. Accordingly, the findings from the multivariable analysis should be regarded as exploratory rather than definitive estimates of effect.

## Figures and Tables

**Table 1:** Demographic, clinical, radiographic, and bronchoscopic characteristics of the study population.

Characteristic	N = 95
Demographic characteristics	
Age (months)	19.0 (14.0–26.0)
Sex	
Female	40 (42.1%)
Male	55 (57.9%)
Clinical characteristics	
Symptom duration (hours)	25.0 (4.0–48.0)
History of aspiration	88 (92.6%)
Sudden coughing episode	89 (93.7%)
Cyanosis/choking history	20 (21.1%)
Wheezing	29 (30.5%)
Dyspnea	16 (16.8%)
Retraction	8 (8.4%)
Oxygen saturation (%)	97.0 (96.0–98.0)
Delayed presentation (>24 h)	50 (52.6%)
Radiographic findings	
Air trapping on CXR	46 (48.4%)
Atelectasis on CXR	7 (7.4%)
Consolidation on CXR	17 (17.9%)
Normal CXR	31 (32.6%)
Thoracic CT performed	11 (11.6%)
Bronchoscopic findings	
Foreign body detected	81 (85.3%)
Negative bronchoscopy	14 (14.7%)
Bronchoscopy localization (*n* = 81)	
Right main bronchus	53 (65.4%)
Left main bronchus	19 (23.5%)
Trachea	4 (4.9%)
Other locations	5 (6.2%)
Foreign body type (*n* = 81)	
Organic	80 (98.8%)
Inorganic	1 (1.2%)
Concomitant infection	13 (13.7%)
Chronic disease	4 (4.2%)

Data are presented as median (IQR) or *n* (%). IQR, interquartile range; CT, computed tomography; CXR, chest radiography.

**Table 2:** Comparison of patients with positive and negative bronchoscopy findings.

Variable	Negative Bronchoscopy (*n* = 14)	Positive Bronchoscopy (*n* = 81)	*p*-Value
Age (months)	15.5 (13.0–24.0)	19.0 (14.0–26.0)	0.300
Symptom duration (hours)	31.0 (5.0–48.0)	25.0 (4.0–48.0)	0.700
Oxygen saturation (%)	97.0 (97.0–98.0)	97.0 (96.0–98.0)	0.500
Male sex	6 (42.9%)	49 (60.5%)	0.300
Delayed presentation (>24 h)	9 (64.3%)	41 (50.6%)	0.400
History of aspiration	14 (100%)	74 (91.4%)	0.600
Sudden coughing episode	13 (92.9%)	76 (93.8%)	0.999
Cyanosis/choking history	6 (42.9%)	14 (17.3%)	0.068
Wheezing	2 (14.3%)	27 (33.3%)	0.200
Dyspnea	0 (0%)	16 (19.8%)	0.120
Retraction	0 (0%)	8 (9.9%)	0.600
Air trapping on CXR	0 (0%)	46 (56.8%)	<0.001
Atelectasis on CXR	0 (0%)	7 (8.6%)	0.600
Consolidation on CXR	1 (7.1%)	16 (19.8%)	0.500
Normal CXR	11 (78.6%)	20 (24.7%)	<0.001
Concomitant infection	0 (0%)	13 (16.0%)	0.200
Chronic disease	1 (7.1%)	3 (3.7%)	0.500

Data are presented as median (IQR) or *n* (%). *p*-values were calculated using the Mann–Whitney U test, Pearson chi-squared test, or Fisher’s exact test, as appropriate. IQR, interquartile range; CXR, chest radiography.

**Table 3:** Univariable and multivariable Firth’s penalized logistic regression analyses for predictors of foreign body detection during bronchoscopy.

Variable	Univariable OR (95% CI)	*p*-Value	Adjusted OR (95% CI)	*p*-Value
Age (per month)	1.00 (0.98–1.03)	0.989	–	–
Symptom duration (per hour)	1.00 (1.00–1.01)	0.802	–	–
Oxygen saturation (per %)	0.89 (0.63–1.15)	0.422	–	–
Male sex	1.99 (0.66–6.30)	0.222	–	–
Delayed presentation (>24 h)	0.59 (0.18–1.81)	0.361	–	–
History of aspiration	0.34 (0.00–3.09)	0.406	–	–
Sudden coughing episode	1.55 (0.15–8.65)	0.664	–	–
Cyanosis/choking history	0.28 (0.09–0.93)	0.038	–	–
Wheezing	2.52 (0.69–13.59)	0.173	1.32 (0.30–7.81)	0.728
Dyspnea	7.31 (0.89–951.17)	0.069	–	–
Retraction	3.35 (0.38–442.52)	0.335	–	–
Concomitant infection	5.72 (0.68–746.46)	0.126	–	–
Chronic disease	0.40 (0.06–4.37)	0.399	–	–
Air trapping on CXR	37.99 (4.79–4914.56)	<0.001	33.80 (4.19–4389.05)	<0.001
Atelectasis on CXR	2.92 (0.32–386.64)	0.406	–	–
Consolidation on CXR	2.27 (0.49–21.78)	0.324	–	–
Normal CXR	0.10 (0.02–0.34)	<0.001	–	–

OR, odds ratio; CI, confidence interval; CXR, chest radiography. The multivariable model included air trapping on chest radiography and wheezing.

## Data Availability

The data presented in this study are available from the corresponding author upon reasonable request. The data are not publicly available because they contain information that could compromise participant privacy and are subject to ethical restrictions.

## Supplementary Materials

The following supporting information can be downloaded at https://www.mdpi.com/article/10.3390/children13070963/s1, Table S1: Clinical and radiographic characteristics of patients with negative bronchoscopy findings; Table S2: Thoracic computed tomography findings according to bronchoscopy results.
