# Supplementary material for: Predictors of Positive Bronchoscopy in Children with Suspected Foreign Body Aspiration: A Retrospective Cross-Sectional Study
Source: Children (Basel). 2026 Jul 21;13(7):963. doi: 10.3390/children13070963 (PMC13406417; doi:10.3390/children13070963)
Supplement: Supplementary file 1 [file children-13-00963-s001.zip › children-4431136-supplementary.pdf]

**Supplementary Table S1. Clinical and Radiographic Characteristics of Patients with Negative Bronchoscopy Findings**

| Variable | Negative Bronchoscopy (*n* = 14) |
|---|---|
| Age (months) | 15.5 (13.0–24.0) |
| Symptom duration (hours) | 31.0 (5.0–48.0) |
| Oxygen saturation (%) | 97.0 (97.0–98.0) |
| Female sex | 8 (57.1%) |
| History of aspiration | 14 (100%) |
| Sudden coughing episode | 13 (92.9%) |
| Cyanosis/choking history | 6 (42.9%) |
| Wheezing | 2 (14.3%) |
| Dyspnea | 0 (0%) |
| Retraction | 0 (0%) |
| Air trapping on CXR | 0 (0%) |
| Atelectasis on CXR | 0 (0%) |
| Consolidation on CXR | 1 (7.1%) |
| Normal CXR | 11 (78.6%) |
| Concomitant infection | 0 (0%) |
| Chronic disease | 1 (7.1%) |

Data are presented as median (IQR) or n (%).
Abbreviations: IQR, interquartile range; CXR, chest radiography.

**Supplementary Table S2. Thoracic Computed Tomography Findings According to Bronchoscopy Results**

| CT Finding | Negative Bronchoscopy (*n* = 2) | Positive Bronchoscopy (*n* = 9) |
|---|---|---|
| Direct foreign body sign | 1 | 6 |
| Air trapping | 0 | 2 |
| Atelectasis | 0 | 3 |
| Consolidation | 1 | 6 |
| Normal CT | 1 | 1 |

Data are presented as n. CT, computed tomography.
Patients may have had more than one CT finding.
